# Sequential acquisition of multi-dimensional heteronuclear chemical shift correlation spectra with ^1^H detection

**DOI:** 10.1038/srep04490

**Published:** 2014-03-27

**Authors:** Peter Bellstedt, Yvonne Ihle, Christoph Wiedemann, Anika Kirschstein, Christian Herbst, Matthias Görlach, Ramadurai Ramachandran

**Affiliations:** 1 Leibniz Institute for Age Research - Fritz Lipmann Institute, Department Biomolecular NMR Spectroscopy, Beutenbergstraße 11, 07745 Jena, Germany; 2 Ubon Ratchathani University, Department of Physics, 34190 Ubon Ratchathani, Thailand; 3 Current address: AiF Projekt GmbH, Tschaikowskistraße 49, 13156 Berlin, Germany.

## Abstract

RF pulse schemes for the simultaneous acquisition of heteronuclear multi-dimensional chemical shift correlation spectra, such as {HA(CA)NH & HA(CACO)NH}, {HA(CA)NH & H(N)CAHA} and {H(N)CAHA & H(CC)NH}, that are commonly employed in the study of moderately-sized protein molecules, have been implemented using dual sequential ^1^H acquisitions in the direct dimension. Such an approach is not only beneficial in terms of the reduction of experimental time as compared to data collection *via* two separate experiments but also facilitates the unambiguous sequential linking of the backbone amino acid residues. The potential of sequential ^1^H data acquisition procedure in the study of RNA is also demonstrated here.

NMR spectroscopy is a powerful technique for the study of biomolecular structure and dynamics, both in solution as well as in the solid state. In protein NMR a variety of multi-dimensional heteronuclear chemical shift correlation experiments are typically used for resonance assignment and for extraction of ^1^H-^1^H distance restraints, *e.g.* HACANH^1^,^2^, HNCAHA^3^,^4^,^5^, HACACONH^2^, HNCOCAHA^4^,^5^, HCCNH^1^,^6^ and ^15^N-edited ^1^H-^1^H NOESY, respectively. These multi-dimensional spectra are based on different magnetisation transfer pathways and are customarily collected individually. As a result, the time for the acquisition of all required data sets can in many cases become exceedingly long. In this context, a variety of techniques are currently being explored for reducing data acquisition times^7^,^8^,^9^,^10^. One of the approaches that has received considerable attention in the study of proteins, both in solution^11^,^12^,^13^,^14^ and in the solid state^15^,^16^,^17^, involves the *simultaneous* collection of different chemical shift correlation spectra. For example, using *dual receivers* with ^1^H *and* ^13^C acquisition in the direct dimension, the simultaneous collection of chemical shift correlation spectra, *e.g.* {HACANH & HACACO} and {HACACO & HACACONH}, has been demonstrated employing both parallel^12^ and sequential^13^ data acquisition procedures. However, as noted already in the literature^11^,^12^, all other aspects being equal, *e.g.* magnetisation transfer and relaxation characteristics, the signal intensities seen in the ^1^H and ^13^C detected data sets would differ because of the difference in the gyromagnetic ratios of the two nuclei. Furthermore, the utility of such experiments is limited as the solution state NMR probes are typically optimised either for ^1^H *or* for ^13^C detection only. In addition, many of the chemical shift correlation experiments of interest do not require dual receivers and instead may involve ^1^H or ^13^C acquisition only. In this context, RF pulse schemes enabling the collection of multi-dimensional data sets with a *single* receiver is of great interest^18^,^19^,^20^,^21^. Recently we have reported RF pulse schemes involving dual sequential ^1^H acquisition with only amide proton detection and making use of dual ^15^N-^13^C mixing steps for achieving protein resonance assignment^22^. While such sequences can be easily adapted to the study of large ^2^H-labeled protein samples, here we present RF pulse schemes that were developed in the context of *moderately* sized proteins. In such systems the relaxation losses during ^15^N-^13^C mixing periods are not expected to be significant even in a fully protonated sample. The pulse sequences presented here make use of the availability of *both* the HA and HN protons and employ only a *single* ^15^N-^13^C mixing step to achieve sequential resonance assignments in protonated protein samples. The efficacy of the approach is experimentally demonstrated by the ‘one-shot' collection of representative protein NMR spectra. We also show that this approach is useful for the study of RNA.

## Results and discussion

### {HA(CA)NH & HA(CACO)NH}

**Figure 1:**
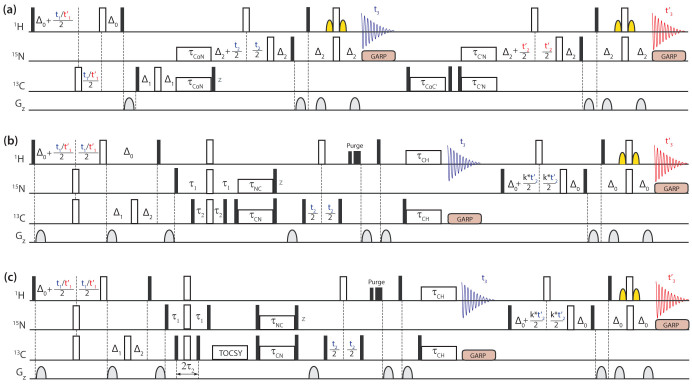
RF pulse schemes for the simultaneous acquisition of (a) 3D {HA(CA)NH & HA(CACO)NH} (b) 3D {H(N)CAHA & HA(CA)NH} and (c) 3D {H(N)CAHA & H(CC)NH} chemical shift correlation spectra of proteins with dual sequential ^1^H acquisitions in the direct dimension. Open and filled rectangles represent 180° and 90° pulses, respectively. Phase cycling is as follows: (a) φ_1_ = *x, − x*; φ_2_ = 8(*x*), 8(−*x*); φ_3_ = 2(*x*), 2(−*x*); φ_4_ = 4(*x*), 4(−*x*); φ_5_ = 2(*y*), 2(−*y*); φ_6_ = 4(*x*), 4(−*x*); φ_R1_ = φ_R2_ = *x*, 2(−*x*), *x*, −*x*, 2(*x*), 2(−*x*), 2(*x*), −*x*, *x*, 2(−*x*), *x*. Gradients with a sine bell amplitude profile were used. Durations and strength with respect to the maximum strength of 50 G/cm are: G_1_ = 1 ms (60%), G_2_ = 1 ms (80%). (b) φ_1_ = *x*, − *x*; φ_2_ = 4(*x*), 4(−*x*); φ_3_ = φ_4_ = 2(*y*), 2(−*y*); φ_5_ = 8(*y*), 8(−*y*); φ_6_ = 4(*y*), 4(−*y*); φ_R1_ = *x*, 2(−*x*), *x*, − *x*, 2(*x*), 2(−*x*), 2(*x*), −*x*, *x*, 2(−*x*), *x*; φ_R2_ = *x*, 2(−*x*), *x*, −*x*, 2(*x*), −*x*; G_1_ = 1 ms (60%), G_2,3_ = 5 ms (60%), G_4_ = 4.4 ms (60%), G_5_ = 1 ms (80%). (c) φ_1_ = *x*, − *x*; φ_2_ = 4(*x*), 4(−*x*); φ_3_ = 16(*y*), 16(−*y*); φ_4_ = 2(*y*), 2(−*y*); φ_5_ = 8(*y*), 8(−*y*); φ_6_ = 4(*y*), 4(−*y*); φ_7_ = 2(*x*), 2(−*x*); φ_R1_ = *x*, 2(−*x*), *x*, −*x*, 2(*x*), 2(−*x*), 2(*x*), −*x*, *x*, 2(−*x*), *x*; φ_R2_ = *x*, 2(−*x), x*, −*x*, 2(*x*), −*x*, *x*, 2(−*x*), *x*, −*x*, 2(*x*), −*x*, −*x*, 2(*x*), −*x*, *x*, 2(−*x*), *x*, −*x*, 2(*x*), −*x*, *x*, 2(−*x*), *x*; G_1_ = 1 ms (60%), G_2,3_ = 5 ms (60%), G_4_ = 4.4 ms (60%), G_5_ = 1 ms (80%).

**Figure 2:**
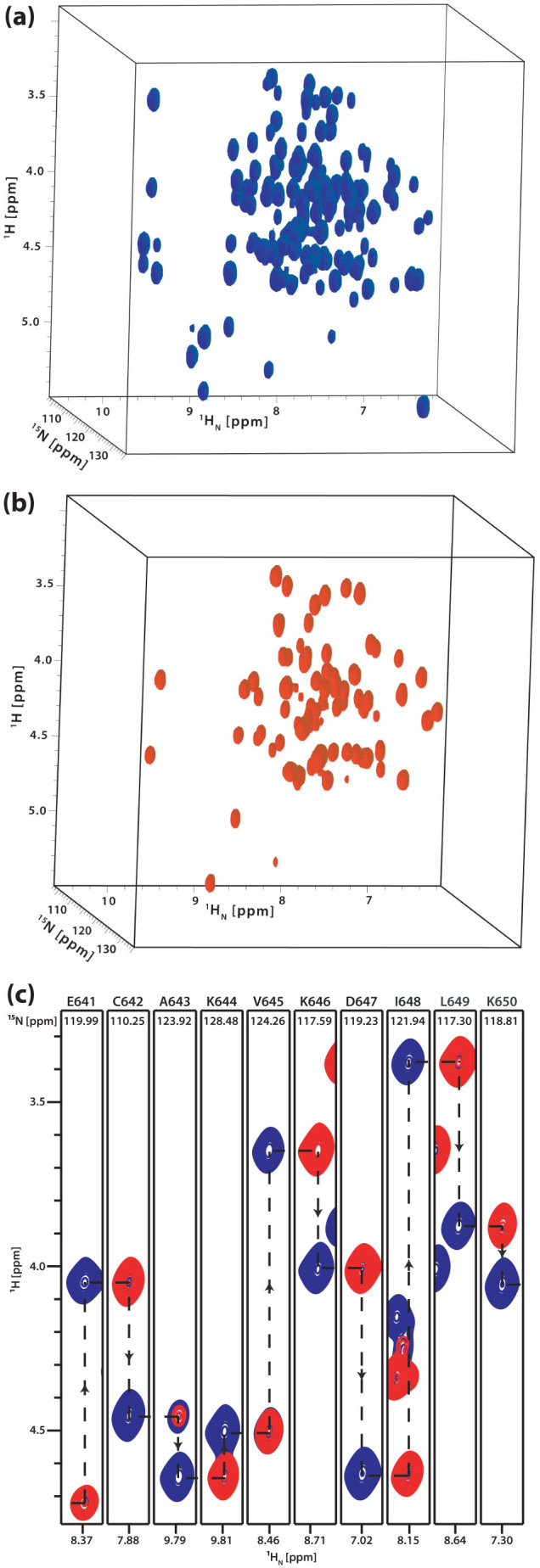
Simultaneously acquired (a) 3D HA(CA)NH and (b) 3D HA(CACO)NH spectra of the MCM C-terminal winged helix domain of *Sulfolobus solfataricus* recorded at 600 MHz with 16 transients per *t*_1_ increment, 41 *t*_1_ increments, 45 *t*_2_ increments, spectral widths of 1559 Hz (^1^H_a_) and 1945 Hz (^15^N) in the indirect dimensions, respectively, a recycle time of 1.0 s and a proton acquisition time of 60 ms in the direct dimension. The total experimental time was 44 h. The AK2-JCH_aniso1_ and AK2-JCaC'_aniso_ sequences were used for ^15^N-^13^C and ^13^C^a^-^13^CO anisotropic cross polarisation, respectively. ^13^C^a^-^13^CO mixing was carried out keeping the ^13^C RF carrier at 115 ppm, with a peak RF power level of ~11 kHz and for a total duration of 17.92 ms by repeating the basic sequence twice (8.96 ms * 2). ^15^N-^13^C mixing was carried out by keeping the ^13^C RF carrier either at 55 ppm or at 175 ppm for achieving band-selective ^15^N-^13^CA and ^15^N-^13^CO cross polarisations for durations of 25 ms and 50 ms (25 ms * 2), respectively. The ^15^N-^13^C mixing sequence with the basic cycle duration of 25 ms was applied employing ^15^N/^13^C peak RF power level of ~3.6 kHz, keeping the ^15^N RF carrier at 121 ppm. The ^1^H RF carrier was kept at 4.3 ppm during *t*_1_ and subsequently switched back to the water position (4.7 ppm). Δ_0,1,2_ = 1.56, 1.56, 2.38 ms were used for INEPT transfers. (c)^1^H_α_-^1^H_N_ spectral cross-sections from the HA(CA)NH (blue) and HA(CACO)NH (red) spectra taken at the ^15^N chemical shifts positions indicated and showing the sequential walk along the backbone residues spanning the region E641-K650.

The triple resonance HACANH experiment is frequently used for sequential resonance assignment of the backbone ^13^C_α_,^15^N,^1^H_N_ and ^1^H_α_ nuclei and involves through-bond magnetisation transfer between the directly coupled nuclei *via* the pathway ^1^H_α_->^13^C_α_->^15^N->^1^H_N_. *Inter*residue cross peaks arising from transfer of magnetisation from the ^13^C_α_ spin of residue (i) to the ^15^N spin of residue of (i + 1), resulting from ^2^J_CaN_ couplings, are observed in this experiment. *Inter*residue cross peaks can usually be distinguished from *intra*residue cross peaks based on their respective signal intensity. However, to achieve unambiguous resonance assignment, the HACACONH experiment involving magnetisation transfers *via* the pathway ^1^H_α_->^13^C_α_->^13^CO->^15^N->^1^H_N_ and leading only to *inter*residue cross peaks is generally carried out in addition. In the HACANH experiment, however, the ^13^C_α_->^15^N magnetisation transfer is the critical step as it relies on weak *intra*residue ^1^J_CaN_ couplings (~11 Hz). Although heteronuclear magnetisation transfers are typically carried out *via* INEPT type transfers, in-phase magnetisation transfers *via* heteronuclear cross polarization schemes have also been successfully used to enhance sensitivity in triple resonance NMR experiments such as in HACANH^1^. Here, we have implemented RF pulse schemes for the sequential collection of different correlation spectra using ^15^N<->^13^C cross polarization schemes. The RF pulse scheme given in Fig. 1a permits the ‘one-shot' acquisition of 3D HA(CA)NH and 3D HA(CACO)NH data sets. The initial transverse ^1^H magnetisation generated by the first 90° pulse is allowed to evolve under its chemical shift during the *t*_1_(HA)/*t*_1_′(HA) period and under the one bond heteronuclear ^13^C-^1^H coupling for a period of 2Δ_0_ to generate antiphase ^1^H magnetisation. The anti-phase ^1^H magnetisation is then converted into antiphase carbon magnetisation by the 90° pulses applied to the two nuclei. The antiphase ^13^C_α_ polarisation is allowed to refocus during the interval 2Δ_1_ to generate (^13^C_α_)^x^ magnetisation and then subjected to a period of ^13^C_α_->^15^N magnetisation exchange *via* the application of a band-selective het-TOCSY mixing sequence. The *residual* ^13^C transverse magnetisation remaining after the ^13^C_α_->^15^N transfer step is flipped to the *z* axis and the ^15^N magnetisation generated after ^13^C_α_->^15^N mixing is allowed to evolve under its chemical shift during the *t*_2_(N) period and transferred to the attached proton *via* an INEPT step and is observed in the *t*_3_ period under ^15^N decoupling to generate the 3D HA(CA)NH spectrum. The WATERGATE sequence^29^ is used for water suppression. After the completion the first ^1^H acquisition, the residual ^13^C_α_ magnetisation is brought to the transverse plane and subjected to ^13^C_α_->^13^CO cross polarisation. This transverse ^13^CO magnetisation is then subjected to a period of ^13^CO->^15^N magnetisation exchange *via* the application of a band-selective het-TOCSY mixing sequence. The resulting transverse ^15^N magnetisation is allowed to evolve during the *t*_2_′ (N) period and then transferred to the attached proton *via* the INEPT procedure. The ^1^H signals are acquired in t_3_′, under ^15^N decoupling to generate the 3D-HA(CACO)NH data. The cross peak intensities observed in the HA(CACO)NH spectrum is dependent on the amount of residual ^13^C_α_ magnetisation present after the ^13^C_α_->^15^N mixing period and hence related to the duration of the mixing period and the performance characteristics of the mixing sequence employed. The residual ^13^C_α_ magnetisation has to be kept along the z axis until the first data acquisition is completed and this may affect the signal intensities observed in the HA(CACO)NH spectrum due to relaxation losses. However, in the systems studied here, significant variation in signal intensities were not observed when the residence time of the ^13^C_α_ magnetisation along the *z* axis was varied over a range of 0–100 ms (Fig. S1). The optimal length of the ^15^N-^13^C het-TOCSY mixing period was found to be ~50 ms (Fig. S2). With this approach we have successfully acquired {3D HA(CA)NH & 3D HA(CACO)NH} spectra of the MCM C-terminal winged helix domain (Fig. 2). Representative spectral cross sections taken from these 3D data sets are given in the supplementary material (Fig. S3, S4) to indicate spectral quality.

### {HA(CA)NH & H(N)CAHA}

The RF pulse scheme given in Fig. 1b allows to simultaneously collect data from both the HA(CA)NH and H(N)CAHA experiments. Here, unlike the case in the RF pulse schemes given in Fig. 1a, the initial transverse magnetisation generated from *both* ^15^N and ^13^C attached protons by the first 90° pulse is allowed to undergo chemical shift evolution during the *t*_1_(HN)/*t*_1_′(HA) period. These evolve under the one bond heteronuclear ^15^N-^1^H and ^13^C-^1^H scalar couplings during the periods 2Δ_0_ and (Δ_0_ + Δ_1_ − Δ_2_), respectively (taking into account the different one bond heteronuclear scalar couplings), to generate the relevant antiphase ^1^H magnetisation. The antiphase ^1^H magnetisation are then converted into the corresponding antiphase nitrogen and carbon magnetisation by the 90° pulses applied to the different nuclei. The antiphase ^15^N and ^13^C polarisation is then allowed to refocus during the interval 2τ_1_ and 2τ_2_ to generate (^15^N/^13^C)^x^ magnetisation and then subjected to ^15^N<->^13^C_α_ magnetisation exchange *via* the application of a band-selective het-TOCSY mixing sequence. Both, the ^15^N and ^13^C transverse magnetisation present after the ^15^N<->^13^C_α_ transfer step is flipped to the *z* axis. First, the data from the ^1^H_N_->^15^N->^13^C_α_->^1^H_α_ pathway [H(N)CAHA] is collected, followed by the acquisition of the signals from the ^1^H_α_->^13^C_α_->^15^N->^1^H_N_ pathway. Sufficient solvent suppression was accomplished by ^1^H *x*- and *y*-purge pulses in combination with gradient pulses^30^ just before the ^13^C-^1^H cross polarisation step and with the ^13^C_α_ magnetisation along the *z* axis. In both data sets, intra- and inter-residue peaks arising, respectively, due to ^1^J_CaN_ and ^2^J_CaN_ couplings are observed.
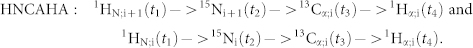

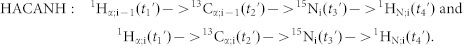


**Figure 3:**
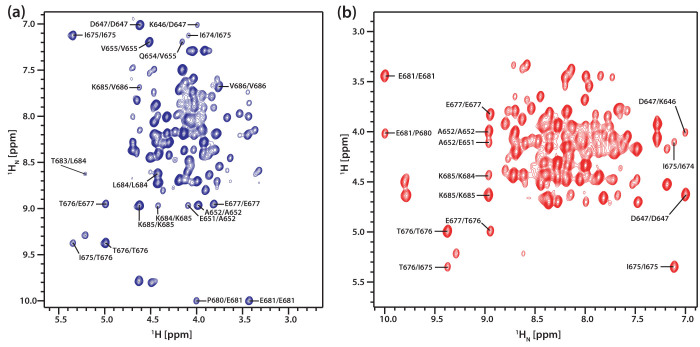
Simultaneously acquired ^1^H-^1^H correlation spectra *via* the (a) 3D H(N)CAHA and (b) 3D HA(CA)NH experiments. These (ω_1_–ω_3_) spectra of the MCM C-terminal winged helix domain of *Sulfolobus solfataricus* recorded at 600 MHz with 16 transients per *t*_1_ increment, 105 *t*_1_ increments, spectral widths in the indirect dimensions of 3598 Hz (^1^H), a recycle time of 1.0 s and a proton acquisition time of 60 ms in the direct dimension. Total experimental time was ~1 h. The AK2-JCH_aniso1_ sequence was used for both ^15^N->^13^CA and ^13^C->^1^H anisotropic cross polarisation transfers. The ^15^N-^13^CA mixing was carried out by keeping the ^13^C RF carrier at 55 ppm, employing ^15^N/^13^C peak RF power level of ~3.6 kHz and for a duration of 50 ms by repeating the basic sequence twice (25 ms * 2). The ^13^C->^1^H het-TOCSY was carried out with one cycle of the AK2-JCH_aniso1_ sequence having a duration of 7.2 ms, employing ^1^H/^13^CA peak RF power level of ~12.5 kHz. The ^1^H RF carrier was kept at 4.7 ppm. The ^1^H RF carrier was kept at 3 ppm during *t*_1_ and subsequently switched back to the water position (4.7 ppm). Δ_0,1,2_ = 2.58, 1.79, 0.79 ms, 2τ_1_ = 2Δ_0_ and 2τ_2_ = 3 ms.

With a *single* ^15^N-^13^C mixing step in the RF pulse sequence (Fig. 1b), the simultaneous collection of H(N)CAHA and HA(CA)NH spectra delivers the chemical shifts of the backbone ^13^C_α;i_,^15^N_i_,^1^H_N;i_ and ^1^H_α;i_ nuclei. Additionally, the (^15^N,^1^H) backbone chemical shifts of the adjacent i+1 residue and the (^13^CA,^1^HA) chemical shifts of the preceding i−1 residue are also obtained. This facilitates the unambiguous linking of three amino acid residues, *i.e.* i-1, i and i+1. With this approach we have successfully acquired a combined data set comprising the HA(CA)NH and H(N)CAHA experiment (Fig. 3). Data collected with a cryoprobe are provided in the supplementary material (Fig. S5, S6) to illustrate the performance of the sequence at lower protein concentrations.

### {H(N)CAHA & H(CC)NH}

**Figure 4:**
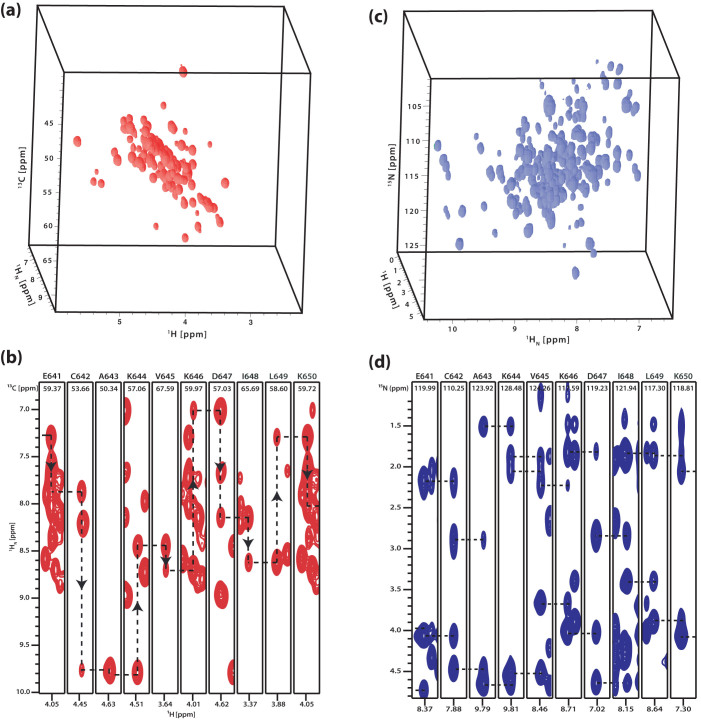
Simultaneously acquired 3D correlation spectra *via* the (a) 3D H(N)CAHA and (b) 3D H(CC)NH experiments. These spectra of the MCM C-terminal winged helix domain of *Sulfolobus solfataricus* recorded at 600 MHz with 16 transients per *t*_1_ increment, 36 *t*_1_ increments, 50 *t*_2_ increments, spectral widths in the indirect dimensions of 3598 Hz (^1^H), 5278 Hz (^13^C), 2639 Hz (^15^N), a recycle time of 1.0 s and a proton acquisition time of 60 ms in the direct dimension. Total experimental time was 42 h. The AK2-JCH_aniso1_ sequence was used for both^15^N->^13^CA and ^13^C->^1^H anisotropic cross polarisation transfers. The ^15^N-^13^CA mixing was carried out by keeping the ^13^C RF carrier at 55 ppm, employing^15^N/^13^C peak RF power level of ~3.6 kHz and for a duration of 50 ms by repeating the basic sequence twice (25 ms * 2). The ^13^C->^1^H het-TOCSY was carried out with one cycle of the AK2-JCH_aniso1_ sequence having a duration of 7.2 ms, employing ^1^H/^13^CA peak RF power level of ~12.5 kHz. Longitudinal ^13^C-^13^C mixing in the aliphatic region was carried out employing the AK2-JCC sequence, with a peak ^13^C RF power level of 10 kHz and for a duration of 9.6 ms by repeating two times the basic cycle of duration 4.8 ms (4.8 ms *2). The RF carrier was kept at 35 ppm during ^13^C-^13^C mixing and at 55 ppm for ^13^CA-^15^N band-selective mixing. The ^1^H RF carrier was kept at 3 ppm during *t*_1_ and subsequently switched back to the water position at 4.7 ppm. Δ_0,1,2_ = 2.58, 1.79, 0.79 ms, 2τ_1_ = 2Δ_0_ and 2τ_2_ = 3 ms were used for INEPT transfers. (c)^1^H_N_-^1^H_α_ spectral cross-sections from the H(N)CAHA spectrum taken at the ^13^C chemical shifts positions indicated and showing the sequential walk along the backbone residues spanning the region E641-K650 (d)^1^H_α_-^1^H_N_ spectral cross-sections from the H(CC)NH spectrum taken at the^15^N chemical shifts positions indicated and showing the connectivities between the adjacent backbone residues.

In addition to resonance assignment of backbone nuclei a modification of the RF pulse scheme given in Fig. 1b allows to simultaneously acquire the 3D H(CC)NH and H(N)CAHA correlation spectra and to obtain chemical shift information on the protein side chain as well as on the backbone nuclei. In a simple HACANH experiment the ^13^C_α_ magnetisation used for ^13^C_α_->^15^N mixing arises only from the magnetisation transfer from directly attached ^1^H_α_ protons. However, the ^13^C_α_ magnetisation in the HCCNH experiment is also generated starting from the side chain protons *via* the ^1^H_sc_->^13^C_sc_->^13^C_α_ magnetisation transfer pathway. This is achieved by introducing a ^13^C-^13^C longitudinal TOCSY mixing period just before the heteronuclear cross polarisation step (Fig. 1c), with the remainder of the pulse sequence essentially the same as in Fig. 1b. Obviously, one can design the RF pulse scheme to obtain either ^1^H or ^13^C side chain chemical shift information. The spectral widths in the indirect dimension in the two data sets can also be *independently* adjusted by appropriate scaling of the *t_2_* (CA)/*t_2_*′ (N) increments and spectral folding in one data set does not lead to resonance overlaps in the other, as the two data sets are effectively independent. As in the case of HACANH experiment, *inter*residue side chain cross peaks arising from transfer of magnetisation from ^13^C_α_ spin of residue (i) to the ^15^N spin of residue of (i + 1) are observed in the HCCNH spectrum. The HCCNH and HNCAHA spectra (Fig. 4) were acquired in one shot *via* the pulse scheme given in Fig. 1c.

The results presented here clearly demonstrate that it is possible to achieve simultaneous acquisition of multidimensional data sets in solution using the sequential data acquisition procedure, akin to recently reported solid state NMR studies of proteins^15^,^16^,^17^. Additionally, the unambiguous sequential linking of backbone nuclei (i − 1, i, i + 1) is achieved in one shot. The basic strategy with sequential data acquisition procedure is that two different experiments leading to correlation spectra arising from different magnetisation transfer pathways are simultaneously started and at a defined intermediate stage the relevant magnetisation belonging to one of the pathways is kept along the *z* axis. Depending on the type of data to be sequentially collected, it can be either ^15^N or the ^13^C nuclei that are to be kept as longitudinal polarisation. After this, magnetisation transfers followed by the first data acquisition are carried out to complete the experiment *via* the first pathway. Subsequently, appropriate magnetisation transfers allow for the second data acquisition to complete the correlation experiment *via* the second pathway. Central to this approach is, that the magnetisation which is stored along the *z* axis for usage in the second experiment should not be disturbed during the completion of the first experiment. The order in which both multi-dimensional data sets are sequentially acquired has to be chosen appropriately in this context. For medium sized molecules, which are the focus of the present study, and with short acquisition times on the order of ~50 ms in the direct dimension, sequential acquisition of correlation spectra do not suffer from significant relaxation losses irrespective of whether ^15^N or ^13^C nuclei are kept along the *z* axis. Although the acquisitions of only a few representative spectra are demonstrated here, the approach outlined can be extended to acquire simultaneously other types of correlation spectra. For example, utilizing the HNCAHA experiment for the sequential assignment of the backbone ^13^C_α_,^15^N,^1^H_N_ and ^1^H_α_ nuclei allow to exploit the residual ^15^N magnetisation after the ^15^N->^13^C_α_ transfer for generating simultaneously a ^15^N edited ^1^H-^1^H NOESY spectrum (Fig. S7, S8, S9). As the application of het-TOCSY mixing schemes over long periods of time may lead to sample heating and hence might pose problems in the study of temperature sensitive samples, one may take recourse to the INEPT procedure to effect ^15^N-^13^C magnetisation transfers. Although the relative merits in the context of sequential data acquisitions are yet to be fully assessed, good quality spectra are obtained by implementing INEPT type transfers^31^ for ^15^N<->^13^C mixing (Fig. S10).

The sequential data acquisition procedure can also be effectively used for simultaneously generating heteronuclear correlation spectra of RNA^32^,^33^. For example, triple resonance NMR experiments such as HCNCH/HCNH are often used for achieving intra- nucleotide correlation of the sugar protons with the base protons. The HCNCH experiment involves through-bond magnetisation transfers between the directly coupled nuclei *via* the pathway ^1^H_1_′->^13^C_1_′->^15^N_1,9_->^13^C_6,8_->^1^H_6,8_ and makes use of ^1^J_CN_ couplings (~12 Hz). The HCNH experiment involves the magnetisation transfer pathway ^1^H_1_′->^3^C_1_′->^15^N_1,9_->^1^H_6,8_ and makes use of ^1^J_CN_ and ^2^J_NH_ couplings. In both experiments, that are typically carried out in D_2_O, the *residual* ^13^C_1_′ magnetisation after the ^ 13^C_1_′->^15^N_1,9_ transfer step can efficiently be exploited to obtain simultaneously COSY/TOCSY data for sugar protons (Fig. S11, S12). For RNA samples dissolved in H_2_O, the through-bond HNCCH experiment is often used for correlating the H3 imino protons of uridines with the H5/H6 base protons *via* the pathway ^1^H_im_->^15^N_im_->^13^C_4_->^13^C_5,6_->^1^H_5,6_. Employing the residual magnetisation after the ^15^N_im_->^13^C_4_ transfer step, one can simultaneously obtain the NOE correlation spectrum of the imino protons in RNA (Fig. S11, S13). The sequential data acquisition strategy presented here may also be combined with other approaches for further reducing the data acquisition time, *e.g.* sparse sampling in the indirect dimension^34^. Furthermore, such dual sequential acquisition procedure may also be applied to collect 3D data with direct ^13^C detection.

## Methods

Uniformly (^13^C,^15^N)-labelled samples of the 82 amino acid MCM C-terminal winged helix domain of *Sulfolobus solfataricus* and the 94 amino acid N-terminal region of human hnRNP C proteins were expressed and purified as reported earlier^23^,^24^. For development, a room temperature triple resonance probe was used and the final protein concentrations were 7 mM and 3 mM, respectively. In addition studies were also undertaken with a cryoprobe utilizing protein samples at lower concentrations (0.8 mM and 1.2 mM, supplementary material). The uniformly (^13^C,^15^N)-labelled RNA (5′-GGCGUUCGCUUAGAACGUC-3′), referred to as BEVSLD5, was prepared as described^25^ and a final concentration of 0.9 mM was used here. Multi-dimensional chemical shift correlation experiments for proteins were carried out with a Bruker 600 MHz narrow-bore Avance III NMR spectrometer equipped with pulse field gradient accessories, pulse shaping units and a triple resonance probe. Sample temperature was set to 303 K. For the BEVSLD5 RNA a triple resonance cryoprobe was used; sample temperature was set to 293 K for experiments on non-exchangeable and 288 K for experiments on exchangeable protons, respectively. Homo- and heteronuclear magnetisation transfers were achieved using amplitude and phase-modulated mixing sequences (AK2-JCH_anisol_, AK2-JCαC'_aniso_:^26^; AK2-JCC^27^). Where required, RF field strength and duration of the mixing period were scaled appropriately. The States procedure^28^ was applied for phase-sensitive detection in the indirect dimensions. Standard phase cycling procedures were employed to select signals arising from desired magnetisation transfer pathways.

## Author Contributions

P.B., Y.I. and R.R. jointly conceived the study and wrote the manuscript. R.R. implemented the idea and collected data. P.B. evaluated data and prepared the figures. Y.I. prepared the RNA sample and analysed data, C.W. prepared the protein sample and analysed data, A.K. and C.H. designed NMR mixing sequences, M.G. corrected the manuscript and supervised the study. All authors reviewed the manuscript.

## Supplementary Material

Supplementary Information Supplementary Material
