# Supplementary material for: Sequential acquisition of multi-dimensional heteronuclear chemical shift correlation spectra with 1H detection
Source: Sci Rep. 2014 Mar 27;4:4490. doi: 10.1038/srep04490 (PMC3967198; doi:10.1038/srep04490)
Supplement: Supplementary Information — Supplementary Material [file srep04490-s1.pdf]

Supplementary material

of the manuscript

Sequential acquisition of multi-dimensional heteronuclear chemical shift correlation spectra with ^{1}H detection

Peter Bellstedt, Yvonne Ihle, Christoph Wiedemann, Anika Kirschstein, Christian Herbst, Matthias Görlach and Ramadurai Ramachandran

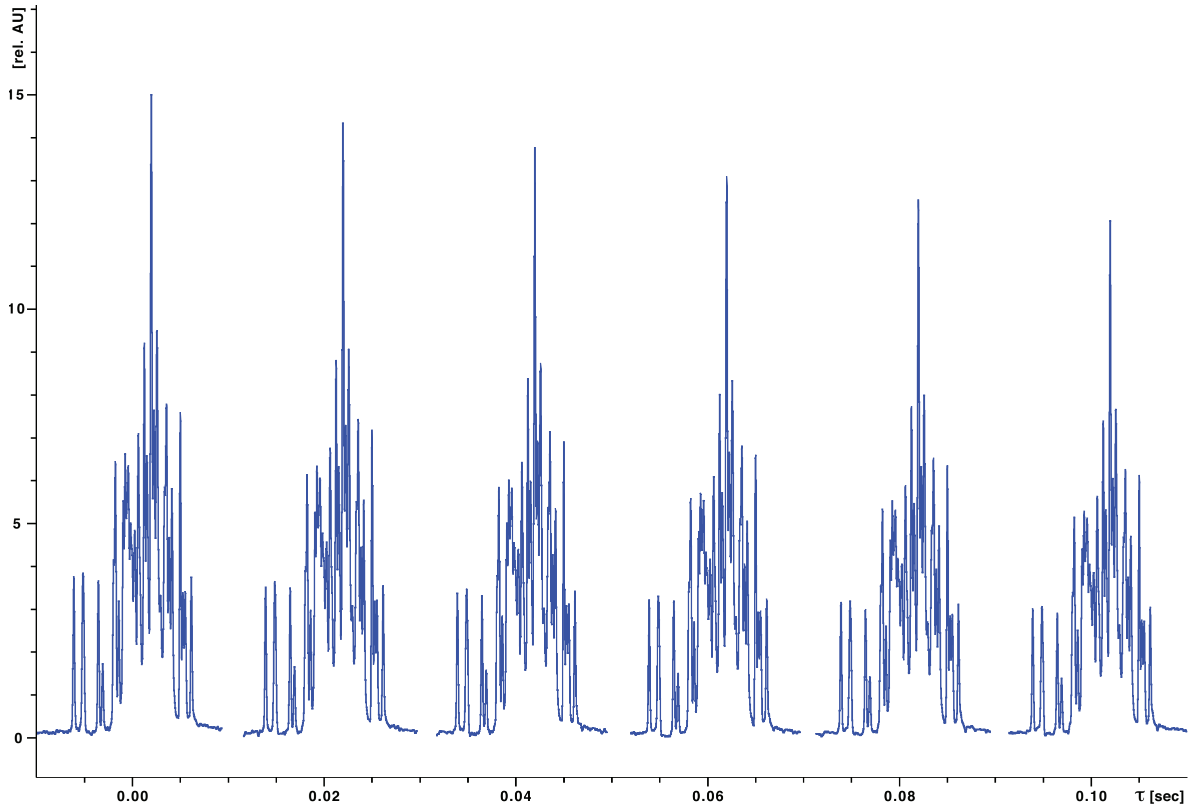

Figure S1. Intensity variations observed for the signals obtained in the first increment using a stand alone HA(CA)NH experiment as a function of the residence time τ of the ^{13}CA magnetisation along the *z* axis. The data were generated with the 7 mM sample of the MCM WH domain using a ^{15}N-^{13}C mixing time of 50 ms, 16 scans, recycle time of 1 s and an acquisition time of 60 ms.

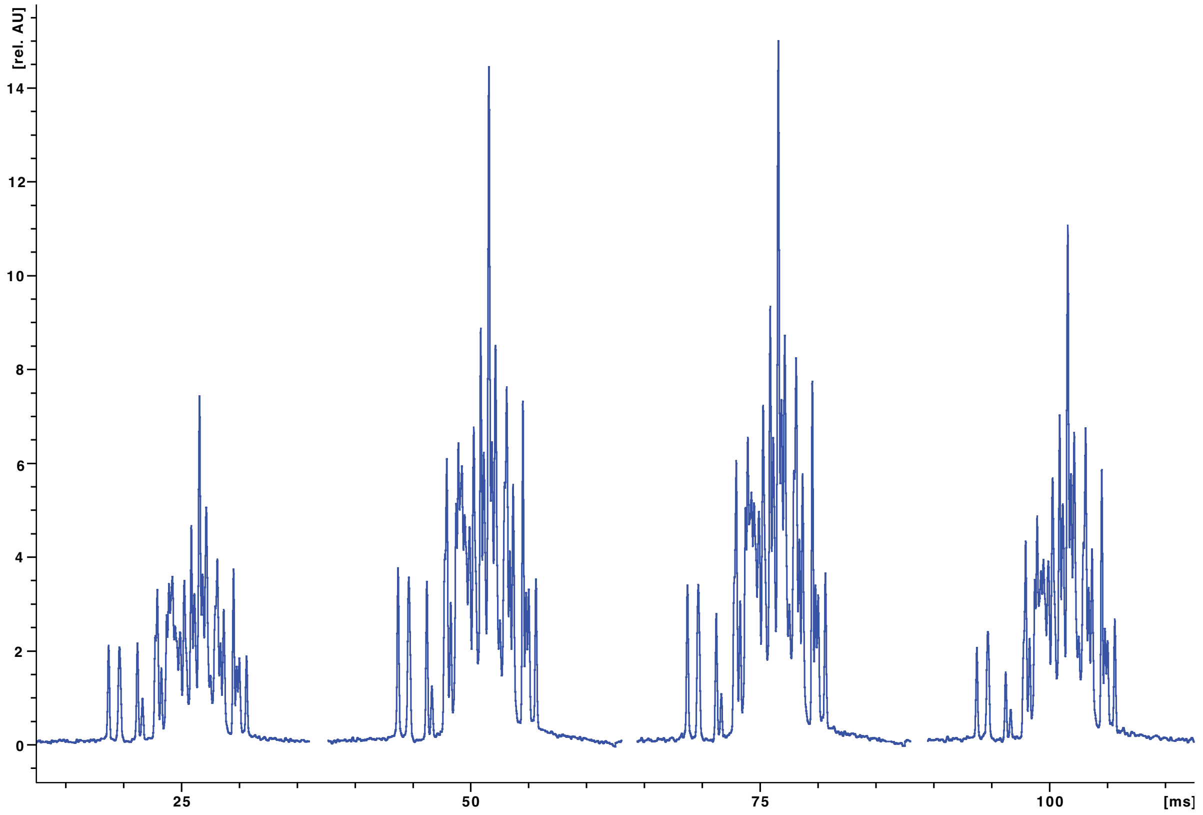

Figure S2. Variations in signal intensities observed in the first increment using a stand alone HA(CA)NH experiment as a function of the ^{15}N-^{13}C mixing time. The data were generated with the 7 mM sample of the MCM WH domain using 16 scans, recycle time of 1 s and an acquisition time of 60 ms.

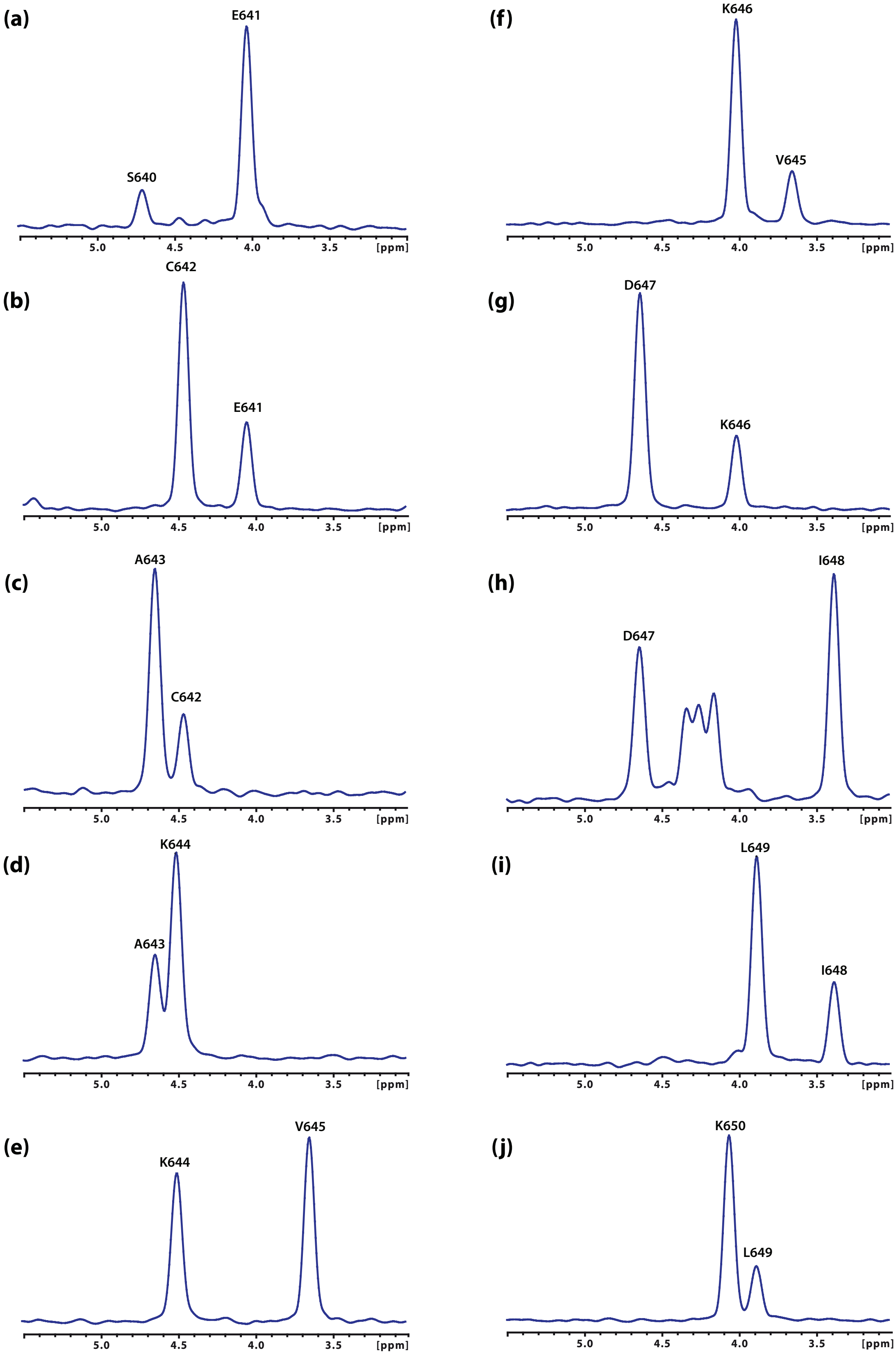

Figure S3. (a-j) 1D spectral cross-sections taken from the 2D HA(CA)NH slices at the amide nitrogen positions of E641-K650 given in Fig. 2c (main text).

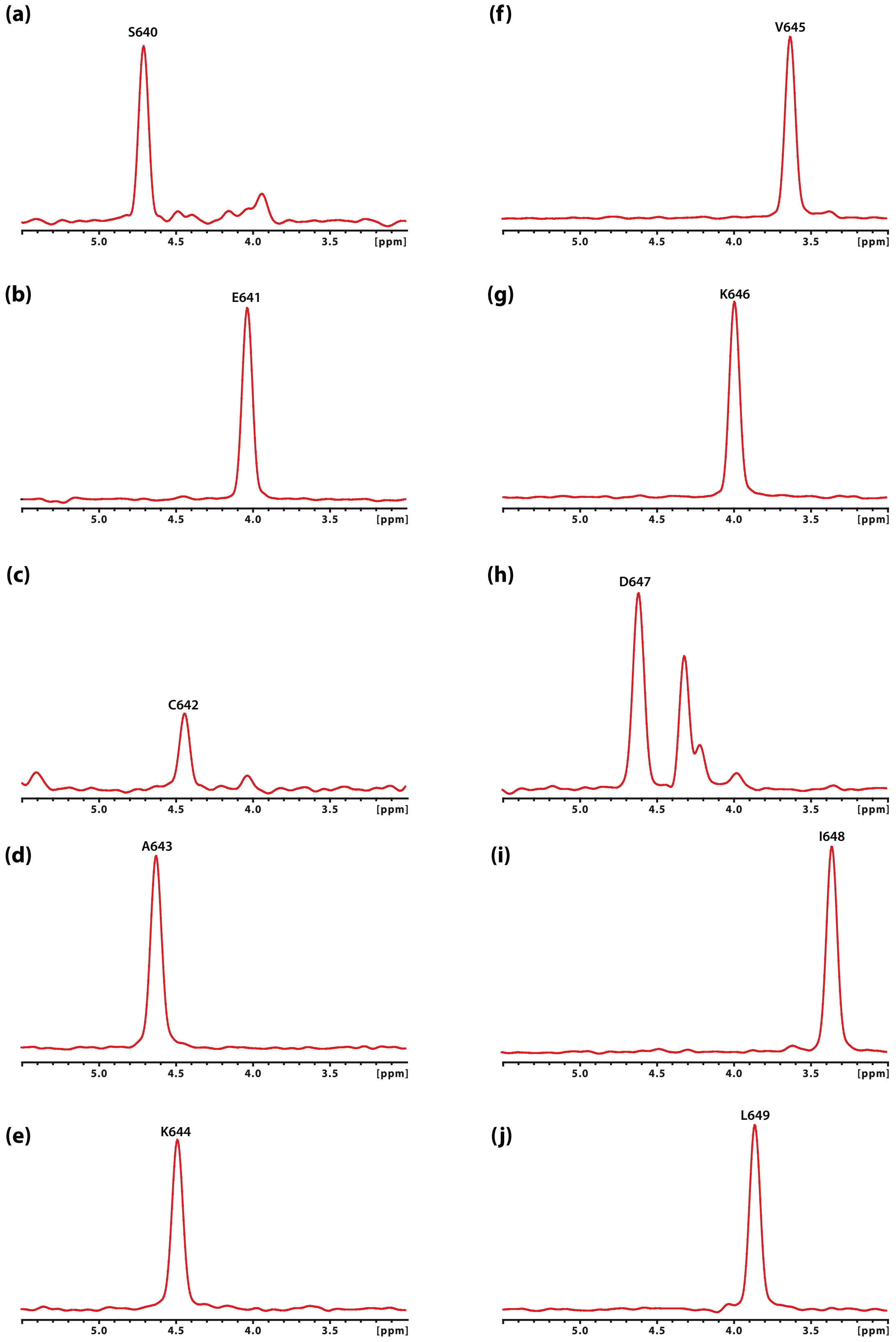

Figure S4. (a-j) 1D spectral cross-sections taken from the 2D HA(CACO)NH slices at the amide nitrogen positions of E641-K650 given in Fig. 2c (main text).

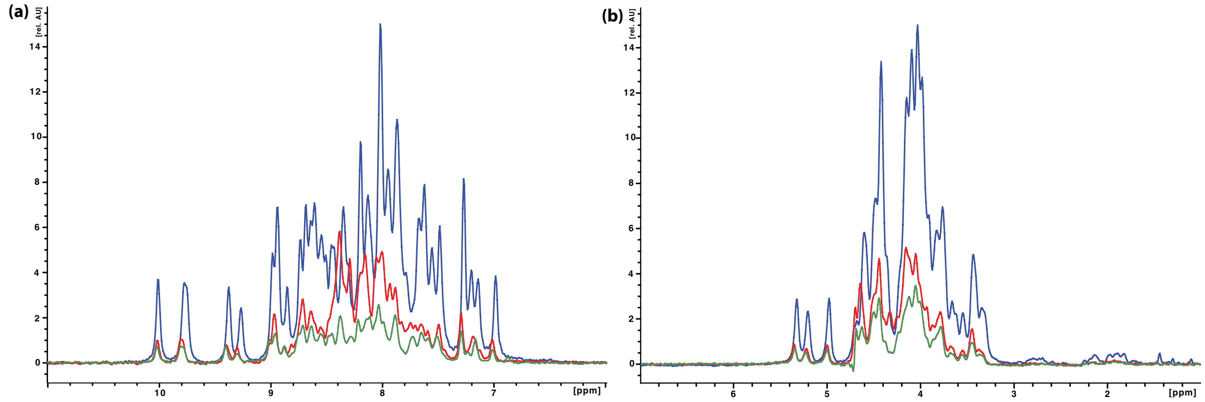

Figure S5. First increments from the sequentially acquired **(a)** HA(CA)NH and **(b)** H(N)CAHA experiments using a cryoprobe with different concentrations (blue: 7 mM, red: 1.2 mM, green: 0.84 mM) of the MCM WH domain and using 16 scans, a recycle time of 1 s and an acquisition time of 60 ms.

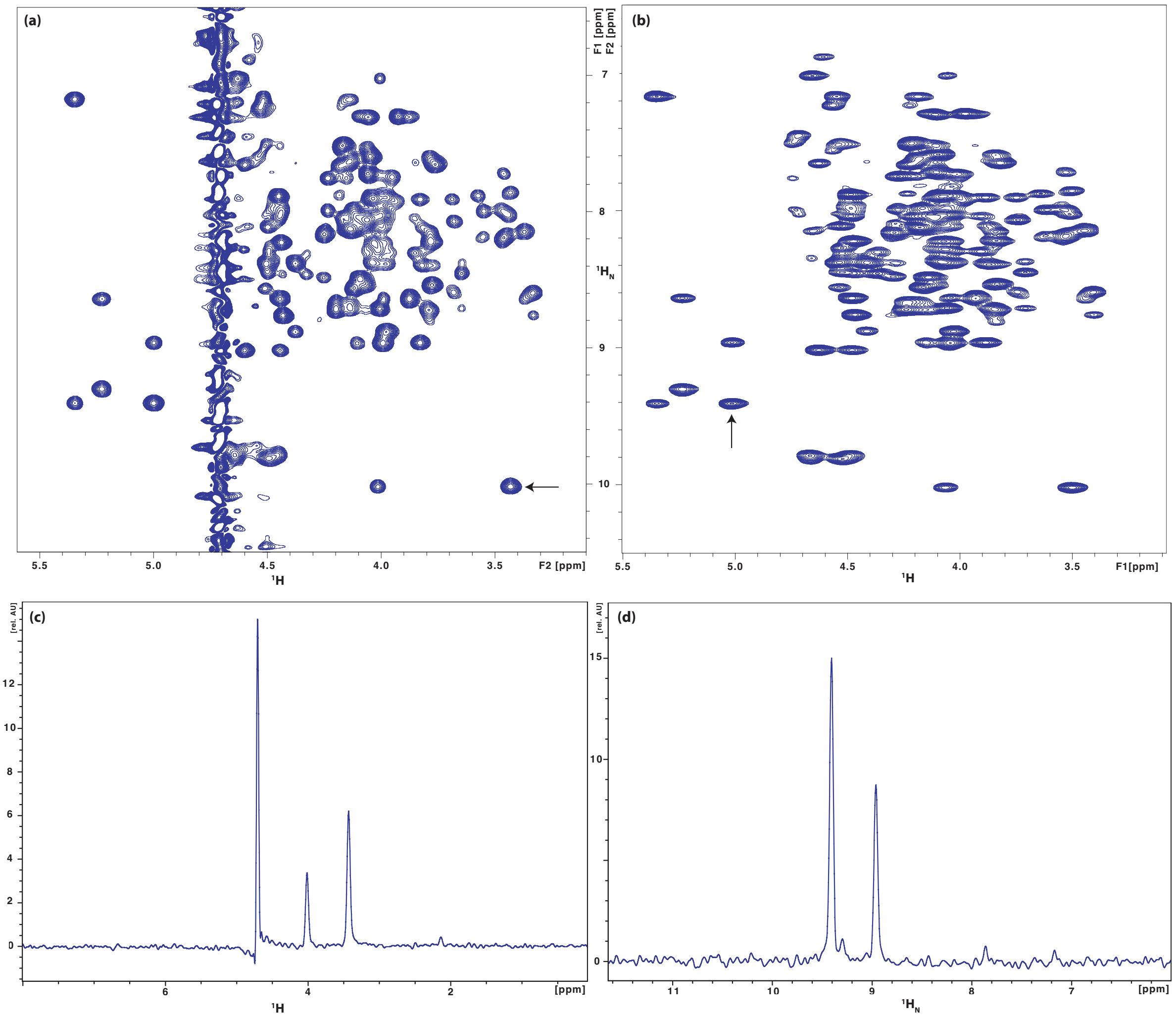

Figure S6. Simultaneously acquired ^1H-^1H correlation spectra via the **(a)** 3D H(N)CAHA and **(b)** HA(CA)NH experiments using a cryoprobe with the MCM WH domain (0.84 mM), 16

transients per t_1 increment, 80 t_1 increments. The ^{15}N-^{13}CA mixing was carried out by keeping the ^{13}C RF carrier at 55 ppm, employing ^{15}N/^{13}C peak RF power level of ~3.125 kHz and for a duration of 57.6 ms by repeating the basic sequence twice (28.8 ms * 2). Other experimental parameters are as given in Fig. 3 (main text). **(c, d)** Spectral cross-sections taken at the positions indicated (arrow) in panel (a) and panel (b), respectively.

{HNCAHA & ^{15}N edited ^{1}H-^{1}H NOESY} experiment:

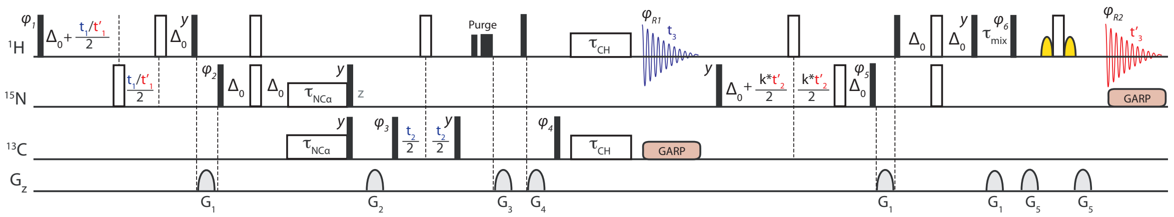

Figure S7. RF pulse scheme for the {HNCAHA & ^{15}N edited ^{1}H-^{1}H NOESY} experiment. Phase cycling is as follows: $\varphi_1 = x, -x$; $\varphi_2 = 4(x), 4(-x)$; $\varphi_3 = 2(y), 2(-y)$; $\varphi_4 = 8(y), 8(-y)$; $\varphi_5 = 2(x), 2(-x)$; $\varphi_6 = 8(y), 8(-y)$; $\varphi_{R1} = \varphi_{R2} = x, 2(-x), x, -x, 2(x), 2(-x), 2(x), -x, x, 2(-x), x$. Durations and strength with respect to the maximum strength of 50 G/cm are: G_1=0.8 ms (80%), G_2=5 ms (50%), G_3=5 ms (40%), G_4=4.4 ms (40%), G_5=0.8 ms (80%).

Description of the {HNCAHA & ^{15}N edited ^{1}H-^{1}H NOESY} experiment (Fig. S7):

The initial transverse ^{1}H magnetisation generated by the first 90° pulse is allowed to evolve under its chemical shift during the t_1/t_1' period and under the one bond heteronuclear ^{15}N-^{1}H scalar coupling for a period of $2\Delta_0$ to generate antiphase ^{1}H magnetisation. The antiphase ^{1}H magnetisation is then converted into antiphase nitrogen magnetisation by the 90° pulses applied to the two nuclei. The antiphase ^{15}N polarisation is allowed to refocus during the interval $2\Delta_0$ to generate $^{15}N^{x}$ magnetisation and then subjected to a period of ^{15}N->$^{13}C_{\alpha}$ magnetisation exchange *via* the application of a ^{15}N->$^{13}C_{\alpha}$ band-selective het-TOCSY mixing sequence. The *residual* ^{15}N transverse magnetisation remaining after the ^{15}N->$^{13}C_{\alpha}$ transfer step is flipped to the *z* axis and the ^{13}C magnetisation generated after ^{15}N->$^{13}C_{\alpha}$ mixing is allowed to evolve under its chemical shift during the t_2 period, transferred to the attached proton *via* an ^{13}C-^{1}H cross polarisation step and observed in the t_3 period under ^{13}C decoupling to generate the 3D HNCAHA spectrum. Solvent suppression was accomplished *via* the application of ^{1}H *x*- and *y*-purge pulses in combination with gradient pulses. After the

completion of the first ^{1}H acquisition, the residual ^{15}N magnetisation is brought to the transverse plane and is allowed to evolve during the t_2' period and then transferred to the attached proton *via* INEPT transfer steps. The in-phase ^{1}H magnetisation at the end of the refocussing period is then flipped to the *z* axis and subjected to a period of NOESY mixing. The longitudinal ^{1}H magnetisation at the end of the NOESY mixing period is brought to the transverse plane and the ^{1}H signals are acquired in t_3' under ^{15}N/^{13}C decoupling. As expected, the signal intensities observed in the NOESY spectrum depend on the amount of residual ^{15}N magnetisation present after the ^{15}N ->$^{13}C_{\alpha}$ mixing period and can be tailored by optimising the duration of the heteronuclear cross polarisation period.

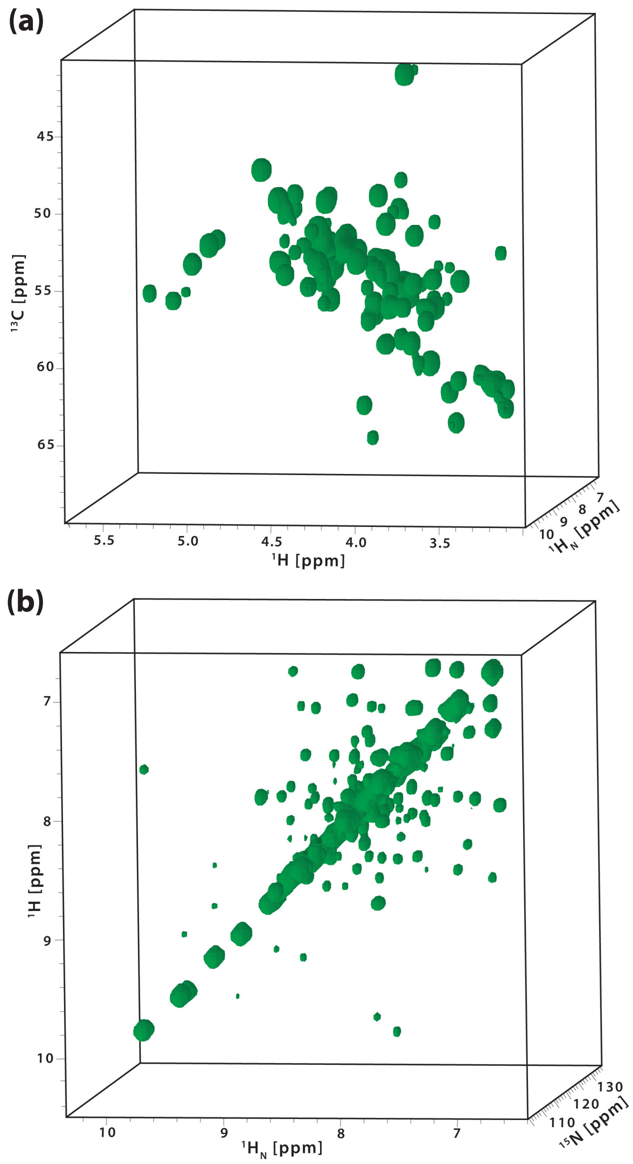

Figure S8. Simultaneously acquired ^{1}H-^{1}H correlation spectra *via* the (a) 3D HNCAHA and (b) 3D ^{15}N-edited $^{1}H_N$-$^{1}H_N$ NOESY experiments. These spectra of the MCM C-terminal winged helix domain of *Sulfolobus solfataricus* recorded at 600 MHz were generated with 16 transients per t_1 increment, 43 t_1 increments, 40 t_2 increments, spectral width in the indirect dimension of 2998 Hz ($^{1}H_N$), 4524 Hz (^{13}C), 2262 Hz (^{15}N), a recycle time of 1.0 s and a proton acquisition in the direct dimension of 60 ms. Total experimental time was 43 h. The AK2-JCH$_{aniso1}$ sequence was used for both ^{15}N->^{13}CA and ^{13}C->^{1}H anisotropic cross polarisation transfers. The ^{15}N-^{13}CA mixing was carried out by keeping the ^{13}C RF carrier at 55 ppm, employing ^{15}N/^{13}C peak RF power level of ~3.6 kHz and for a duration of 50 ms by repeating the basic sequence twice (25 ms * 2). The ^{13}C->^{1}H het- TOCSY was carried out with one cycle of the AK2-JCH$_{aniso1}$ sequence having a duration

of 7.2 ms, employing $^{1}H/^{13}CA$ peak RF power level of ~12.5 kHz. The ^{1}H RF carrier was kept at 8 ppm during t_1 and switched back to the water position (4.7 ppm) after that. De- and rephasing periods of ~5 ms (^{15}N-^{1}H) and ~3 ms (^{13}C-^{1}H) were used for INEPT transfers. The NOESY spectrum was collected under ^{15}N decoupling with a mixing time of 100 ms.

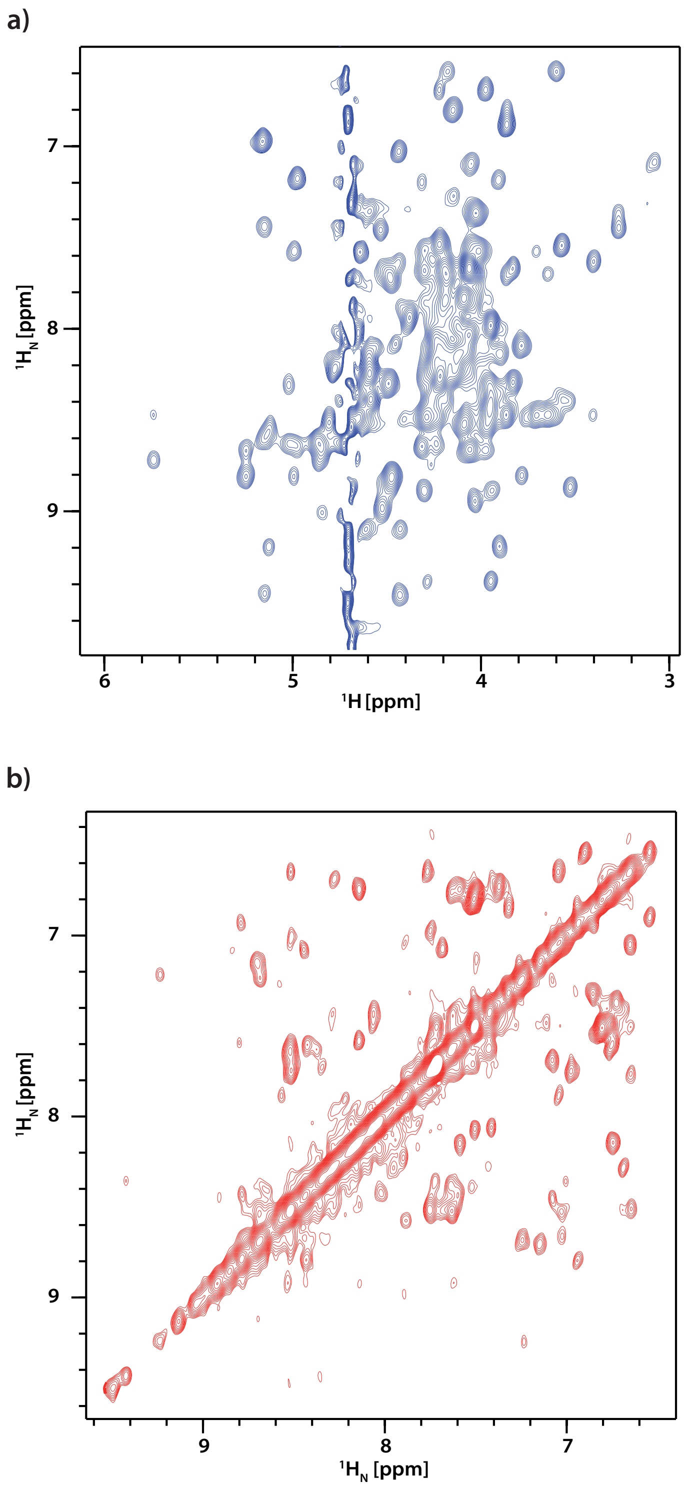

Figure S9. Simultaneously acquired ^{1}H-^{1}H correlation spectra *via* the 3D HNCAHA (a) and 3D ^{15}N-edited ^{1}H-^{1}H NOESY (b) experiments. These (w_1-w_3) spectra of the RNA-binding domain of human hnRNP C proteins recorded at 600 MHz were generated with 80 transients per t_1 increment, 50 t_1 increments, spectral width in the indirect $^{1}H_N$ dimension of 2398 Hz, a recycle time of 1.0 s and a proton acquisition in the direct dimension of 60 ms. Overall experimental time was ~3 h. The AK2-JCH$_{aniso1}$ sequence was used for both ^{15}N->^{13}CA and ^{13}C->^{1}H anisotropic cross polarisation transfers. The ^{15}N-^{13}CA mixing was carried out by keeping the ^{13}C RF carrier at 55 ppm, employing $^{15}N/^{13}C$ peak RF power level of ~3.6 kHz and for a duration of 50 ms by repeating the basic sequence twice (25 ms * 2). The ^{13}C->^{1}H het-TOCSY was carried out with one cycle of the AK2-JCH$_{aniso1}$ sequence having a duration of 7.2 ms, employing $^{1}H/^{13}CA$ peak RF power level of ~12.5 kHz. The ^{1}H RF carrier was kept at 8 ppm

during t_1 and switched back to the water position (4.7 ppm) after that. ^{15}N-^{1}H and ^{13}C-^{1}H dephasing and rephasing periods of ~4.8 ms and 3.1 ms, respectively, were used for INEPT transfers. The NOESY spectrum was collected with a mixing time of 200 ms.

INEPT version of the {H(N)CAHA & HA(CA)NH} experiment:

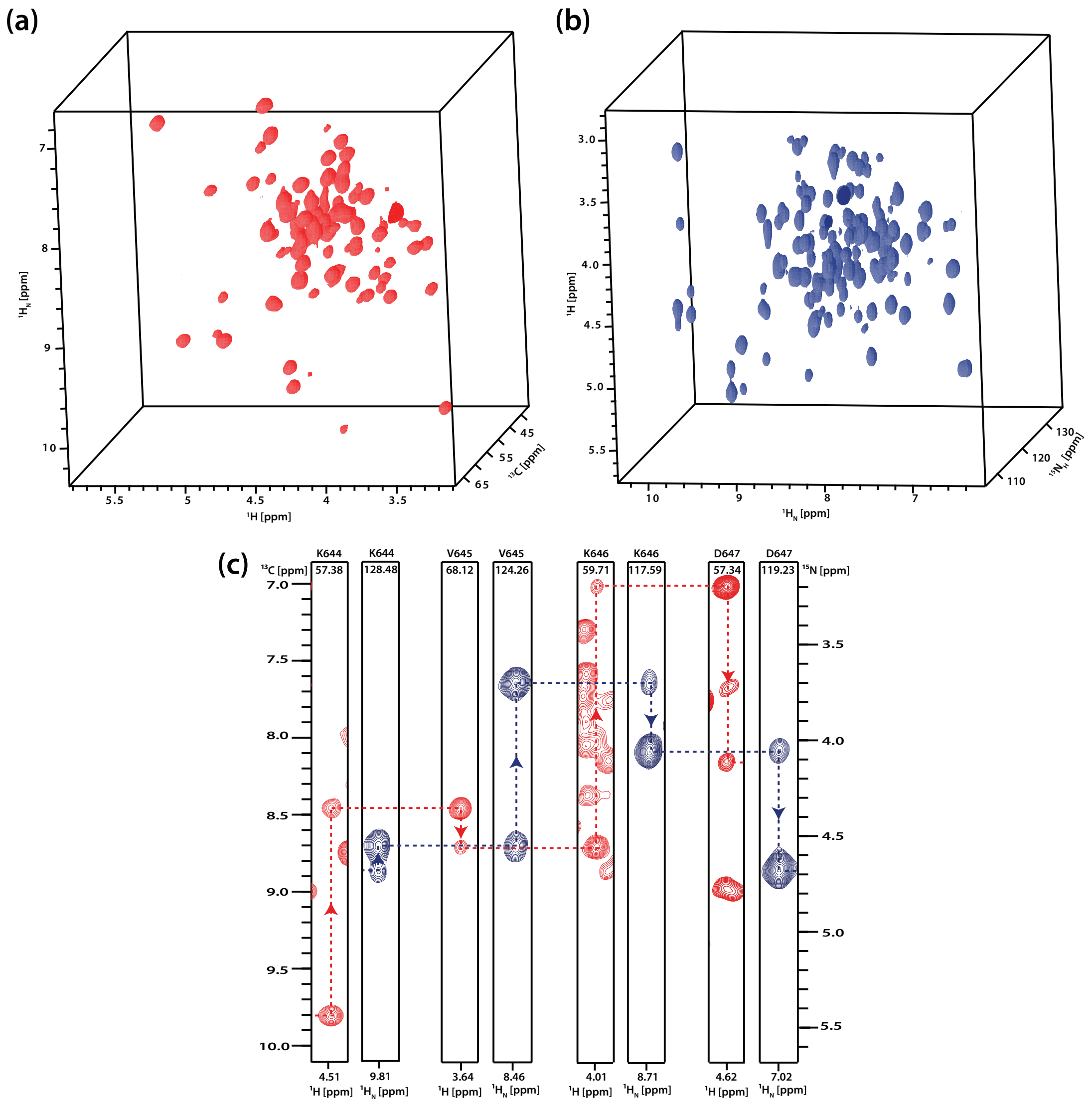

Figure S10. Simultaneously acquired **(a)** 3D H(N)CAHA and **(b)** 3D HA(CA)NH correlation spectra using the INEPT procedure for ^{15}N-^{13}C mixing. The RF pulse scheme used is essentially the same as given in Fig. 1b (main text) except that the 15N-13C het-TOCSY mixing sequence was replaced by the sandwich $\{\tau\text{-}(180)^{13C,15N}\text{-}\tau\text{-}(90)^{13C,15N}\text{-}\tau\text{-}(180)^{13C,15N}\text{-}\tau\}$ along with simultaneous ^{1}H decoupling during the INEPT transfer step (τ=12.5 ms). The RF

pulses were phase cycled appropriately to select the signals *via* the coherence transfer pathways of interest.

These spectra of the MCM C-terminal winged helix domain of *Sulfolobus solfataricus* recorded at 600 MHz with 16 transients per t_1 increment, 36 t_1 increments, 50 t_2 increments, spectral widths in the indirect dimensions of 3598 Hz (^{1}H), 4825 Hz (^{13}C), 2413 Hz (^{15}N), a recycle time of 1.0 s and a proton acquisition time of 60 ms in the direct dimension. The overall experimental time was 42 h. **(c)** ^{1}H-^{1}H spectral cross-sections from the H(N)CAHA and HA(CA)NH spectra taken at the ^{13}C and ^{15}N chemical shifts positions indicated and showing the connectivities between the adjacent backbone residues spanning the region E644- K647.

RF pulse schemes for RNA experiments:

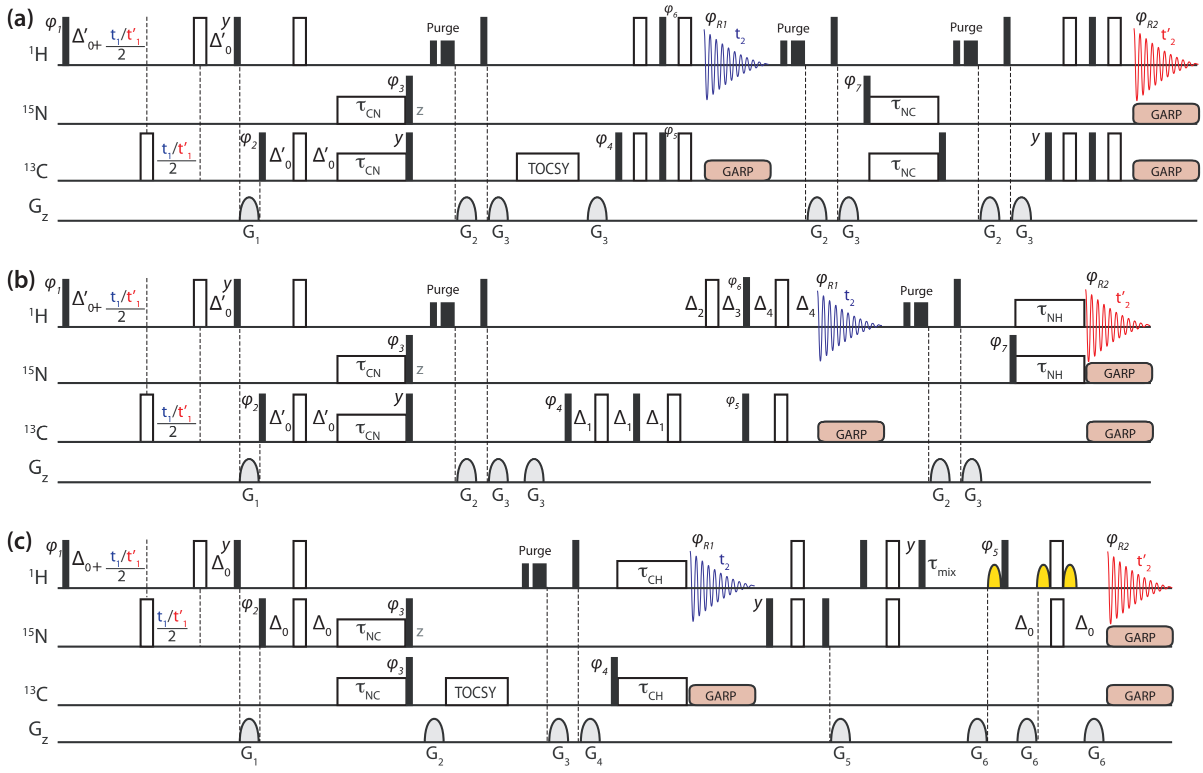

Figure S11. RF pulse schemes for the acquisition of sugar and base proton correlations [H(CNC)H (a), H(CN)H (b)] together with TOCSY (**a**) and COSY correlations (**b**) of the sugar protons. (**c**) RF pulse scheme to correlate the imino protons of the base with the aromatic protons together with the NOE correlations of the imino protons. Phase cycling is as

follows: (a, b) $\varphi_1=x, -x;$ $\varphi_2=4(x), 4(-x);$ $\varphi_3=2(y), 2(-y);$ $\varphi_4=8(y), 8(-y);$ $\varphi_5=16(x), 16(-x);$ $\varphi_6=2(y), 2(-y);$ $\varphi_7=8(y), 8(-y);$ $\varphi_{R1}=x, 2(-x), x, -x, 2(x), 2(-x), 2(x), -x, x, 2(-x), x, -x, 2(x), -x, x, 2(-x), x, x, 2(-x), x, -x, 2(x), -x;$ $\varphi_{R2}=x, 2(-x), x, -x, 2(x), 2(-x), 2(x), -x, x, 2(-x), x$. Durations and strength with respect to the maximum strength of 50 G/cm are: G_1=1 ms (60%), G_2=5 ms (70%), G_3=4.4 ms (70%). (c) $\varphi_1=x, -x;$ $\varphi_2=4(x), 4(-x);$ $\varphi_3=2(y), 2(-y);$ $\varphi_4 = \varphi_5 = 8(y), 8(-y);$ $\varphi_{R1}=\varphi_{R2}=x, 2(-x), x, -x, 2(x), 2(-x), 2(x), -x, x, 2(-x), x;$ G_1=0.7 ms (80%), G_2=4 ms (80%), G_3=4 ms (60%), G_4=2 ms (60%), G_5=0.7 ms (60%), G_6=0.7 ms (80%).

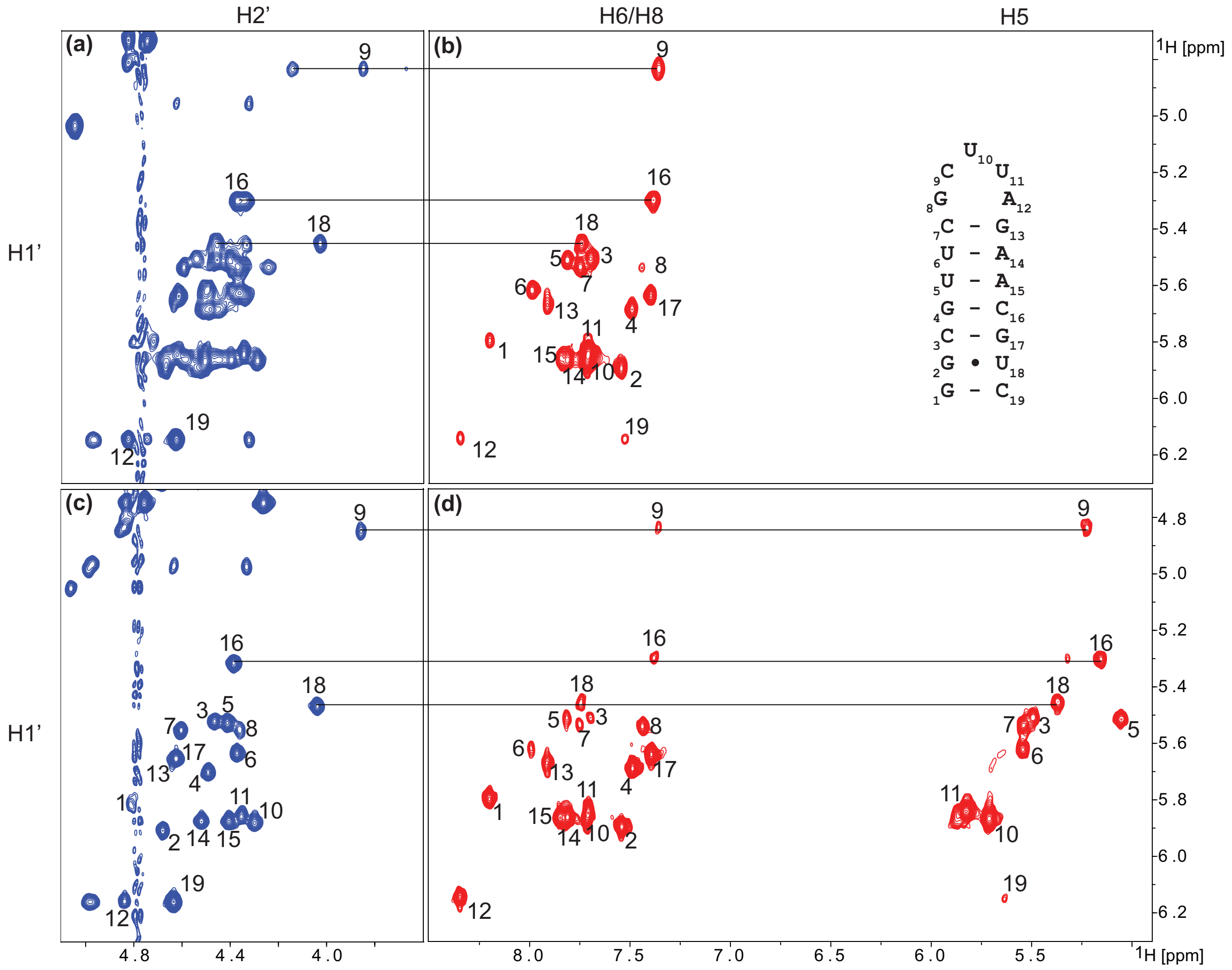

Figure S12. Simultaneously acquired **(a)** sugar protons ^{1}H-^{1}H correlation spectra and **(b)** sugar-base [through bond, H(CNC)H] of the 0.9 mM BEVSLD5 RNA (see material & methods section of main text for reference). These spectra were sequentially recorded at 600 MHz with 96 transients per t_1 increment, 40 t_1 increments, spectral width in the indirect ^{1}H dimension of 1559 Hz, a recycle time of 2.0 s and a proton acquisition in the direct dimension of 60 ms. Total experimental time was ~5 h. The AK2-JCH$_{aniso1}$ sequence was used for ^{15}N-^{13}C anisotropic cross polarisation transfers. The ^{15}N-^{13}C$_1$' and ^{15}N-^{13}C$_{6,8}$ mixings were carried

out by keeping the ^{13}C RF carrier at 90 and 135 ppm, respectively, ^{15}N RF carrier at 155 ppm, employing $^{15}N/^{13}C$ peak RF power level of ~3.125 kHz and for a duration of 57.6 ms (^{15}N-$^{13}C_1$') and 86.4ms (^{15}N-$^{13}C_{6,8}$) by repeating the basic sequence of 28.8 ms duration two or three times. Longitudinal ^{13}C-^{13}C mixing in the sugar region was carried out employing AK2-JCC sequence, with a peak ^{13}C RF power level of 10 kHz and for a duration of 9.6 ms by repeating two times the basic cycle of duration 4.8 ms (4.8 ms *2). The RF carrier was kept at 75 ppm during ^{13}C-^{13}C mixing. The ^1H RF carrier was kept at 5.3 ppm during t_1 and subsequently switched back to the water position (4.7 ppm). ^{13}C-^1H de- and rephasing periods of ~3.1 ms, respectively, were used for INEPT transfers. Simultaneously acquired **(c)** sugar protons ^1H-^1H correlation spectra and **(d)** sugar-base (HCNH) spectra of the same RNA sample. These spectra were sequentially recorded at 600 MHz with 96 transients per t_1 increment, 64 t_1 increments, spectral width in the indirect ^1H dimension of 1919 Hz, a recycle time of 2.0 s and a proton acquisition time of 60 ms in the direct dimension. Total experimental time was ~8 h. The AK2-JCH$_{aniso1}$ sequence was used for ^{15}N-^{13}C anisotropic cross polarisation transfers. The ^{15}N-$^{13}C_1$' mixing was carried out by keeping the ^{13}C and ^{15}N RF carriers at 90 and 155 ppm, respectively, employing $^{15}N/^{13}C$ peak RF power level of ~3.125 kHz and for a duration of 57.6 ms by repeating the basic sequence of 28.8 ms duration twice. COSY type ^{13}C-^{13}C mixing of the sugar carbons was carried out employing the delay values: Δ_1= 3.25ms, Δ2= 2.14ms, Δ3= 1.11ms, Δ_4= 1.52 ms. The RF carrier was kept at 75 ppm during ^{13}C-^{13}C mixing. The ^{15}N-^1H het-TOCSY mixing was carried out using the AK2-JCH$_{aniso1}$ cross-polarisation sequence by keeping the ^1H and ^{15}N RF carriers at 7 and 155 ppm, respectively, employing $^{15}N/^1H$ peak RF power level of ~3.125 kHz and for a duration of 86.4 ms by repeating the basic sequence of 28.8 ms duration twice.

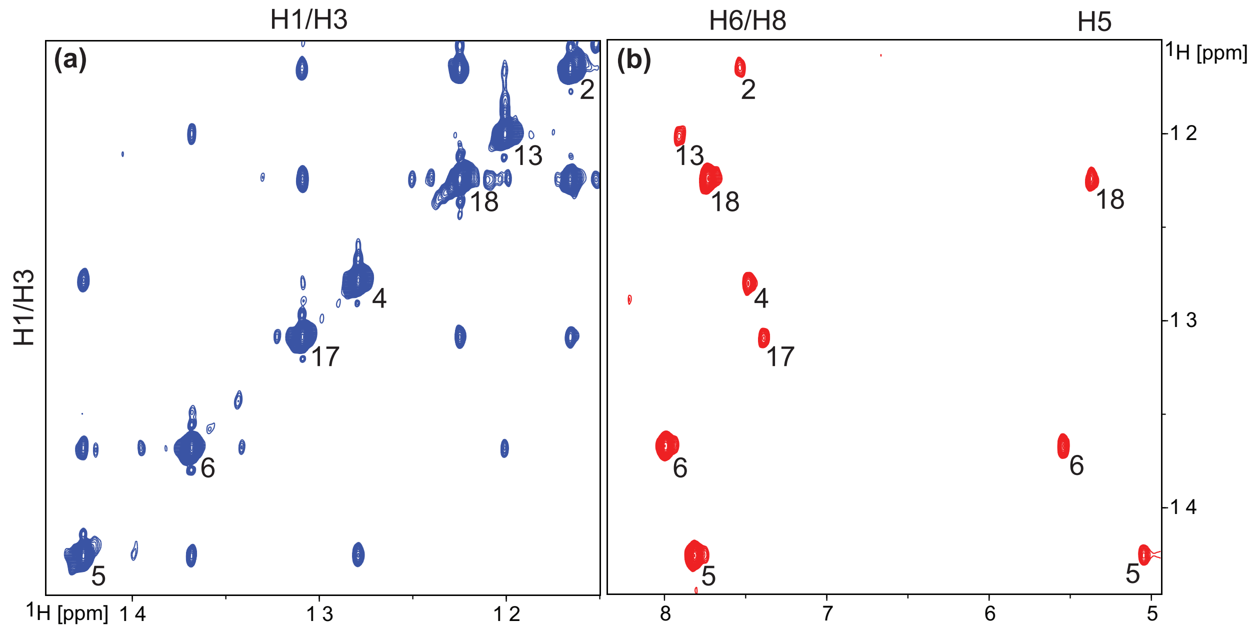

Figure S13. Simultaneously acquired **(a)** NOE correlations of the imino protons and **(b)** imino-base proton correlations [through bond, H(NCC)H] of the 0.9 mM BEVSLD5 RNA. These spectra were sequentially recorded at 600 MHz with 224 transients per t_1 increment, 100 t_1 increments, spectral width in the indirect ^{1}H dimension of 3000 Hz, a recycle time of 2.0 s and a proton acquisition in the direct dimension of 70 ms. Total experimental time was ~25 h. The AK2-JCH$_{aniso1}$ sequence was used for ^{15}N-^{13}C anisotropic cross polarisation transfer, keeping the ^{13}C and ^{15}N RF carriers at 165 and 166 ppm, respectively, employing ^{15}N/^{13}C peak RF power level of ~3.125 kHz and for a duration of 28.8 ms by applying the basic sequence of 28.8 ms duration once. Longitudinal ^{13}C-^{13}C mixing in the aromatic region was carried out employing AK2-JCC sequence, with a peak ^{13}C RF power level of 10 kHz and for a duration of 9.6 ms by repeating two times the basic cycle of duration 4.8 ms (4.8 ms *2). The RF carrier was kept at 138 ppm during ^{13}C-^{13}C mixing. The ^{1}H RF carrier was kept at 12.5 ppm during t_1 and subsequently switched back to the water position (4.7 ppm). The ^{13}C->^{1}H het-TOCSY was carried out with one cycle of the AK2-JCH$_{aniso1}$ sequence having a duration of 7.2 ms, employing ^{1}H/^{13}CA peak RF power level of ~12.5 kHz. An NOE mixing time of 200 ms was employed.
